# It’s QuizTime! The impact of web-based microlearning quizzes on guideline-concordant antibiotic duration for pediatric acute otitis media

**DOI:** 10.1017/ash.2025.10160

**Published:** 2025-11-03

**Authors:** Brittany J. Lehrer, Meng Xu, Lori A. Deitte, Ritu Banerjee, Sophie E. Katz

**Affiliations:** 1 Division of Infectious Diseases, Department of Pediatrics, Vanderbilt University Medical Center, Nashville, TN, USA; 2 Department of Biostatistics, Vanderbilt University Medical Center, Nashville, TN, USA; 3 Department of Radiology and Radiological Sciences, Vanderbilt University Medical Center, Nashville, TN, USA

## Abstract

We evaluated the impact of a web-based quiz on prescribing guideline-concordant antibiotic duration for pediatric acute otitis media. Adjusted for baseline prescribing, participants increased mean guideline-concordant prescribing by 9.6% compared to nonparticipants; those who took an enhanced quiz increased 17.1%. QuizTime may be a low-effort intervention to increase stewardship education.

## Introduction

Most children receive 10-day antibiotic durations for acute otitis media (AOM) despite the American Academy of Pediatrics’ (AAP) 2013 and associated institutional recommendations for 5 – 7 days in children aged ≥ 2 years.^1–4^ This discrepancy may be due to lack of guideline awareness. QuizTime is a web-based application that delivers daily quiz questions to learners’ mobile devices, using Test-Enhanced Learning Theory to augment medical education.^5^ This theory suggests that testing not only assesses knowledge but also enhances retention.^6^ We sought to evaluate the impact of participating in a basic QuizTime intervention, with or without a subsequent enhanced intervention, on prescribing guideline-concordant antibiotic durations for pediatric AOM.

## Methods

### Study design, intervention, setting, population, and outcome

We conducted a prospective cohort study of clinicians who prescribed antibiotics for AOM to children <18 years old at a Vanderbilt University Medical Center (VUMC) clinic or emergency department from 7/1/2021 – 9/30/2023. The study had two intervention groups—basic or enhanced. The basic intervention consisted of 10 case-based, multiple-choice questions addressing guideline-concordant treatment of AOM, community-acquired pneumonia (CAP), urinary tract infections (UTI), and when to test for Group A streptococcal (GAS) pharyngitis (Supplemental Methods). Participants received one question daily on their mobile devices for 10 consecutive weekdays starting 7/1/2022 and had 48 hours to respond to each question. Correct answers and learning objectives were shown immediately after submission (Supplemental Figure 1). Half of the participants who enrolled in the basic intervention were randomly selected and invited to enroll in the enhanced intervention five months later (Supplemental Figure 2). Participants in the enhanced intervention received five additional questions beginning 11/1/2022. These questions were those that were most frequently answered incorrectly (in aggregate) during the basic intervention (Supplemental Methods). Clinicians were excluded if they: prescribed antibiotics in subspeciality clinics; prescribed antibiotics only from 7/1 – 7/14/2021 (basic intervention washout period) or 11/1 – 11/7/2021 (enhanced intervention washout period); or provided ≤ 3 antibiotic prescriptions for AOM during the study period. The study outcome was the percentage of encounters with a diagnosis of AOM and age-appropriate antibiotic duration, as defined by the AAP guidelines (< 2 yr = 10 d, 2 – 5 yr = 7 d, 6+ years = 5 – 7 d).^1^ We focused on duration because local data^7^ and prior studies^3,7^ demonstrate antibiotic choice for AOM is generally guideline-concordant. We evaluated prescriber-level change from baseline in percentage of guideline-concordant prescriptions and compared the average aggregate change for each intervention group to the nonparticipant group.

### Data source

Antibiotic prescription date, clinic type, agent, duration, clinician type, and patient age, sex, race, and insurance type were extracted from electronic health records (Epic, Verona, WI). These data were obtained for all participants and nonparticipants as part of routine stewardship monitoring.

Antibiotic indication of AOM was determined by (in order of hierarchy): manual entry of AOM order indication (required with order entry beginning 5/18/2022), *International Classification of Disease, 10* ^*th*^ *modification* (ICD-10) code for AOM associated with the prescription (H65.XX or H66.XX), or ICD-10 code for AOM among the first three assigned visit diagnoses.

### Recruitment

We emailed medical directors across all VUMC-associated clinics and emergency departments to introduce the study and encourage clinician participation. Enrollees were offered Continuing Medical Education and Maintenance of Certification Part 2 credit. Participants who completed the enhanced intervention received a $20 gift card. VUMC Institutional Review Board approved the study.

### Time periods

Antibiotic prescribing data were collected from all participants and nonparticipants throughout the following periods: Baseline Period (7/1/2021 – 6/30/2022), Period 1 (7/1/2022 – 10/31/2022), and Period 2 (11/1/2022 – 3/31/2023). A postquiz period without further interventions occurred from 4/1/2023 – 9/30/2023 to evaluate sustainability (Supplemental Figure 2).

### Statistical analysis

**Figure 1.**
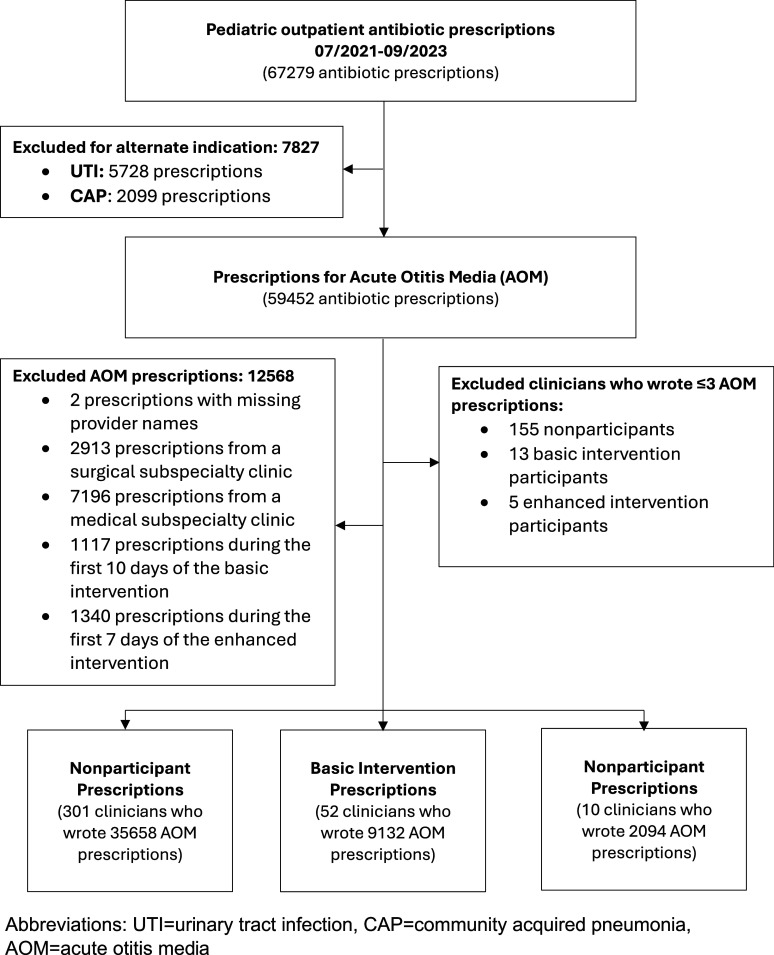
Strengthning the reporting of observational studies in epidemiology (strobe) diagram.

We compared the proportion of guideline-concordant antibiotic prescriptions for treatment duration at the provider-level using a Kruskal-Wallis test with a statistical significance level of .05. Additionally, we used a multivariate linear logistic regression model to assess changes in guideline-concordant prescribing from baseline across the participant and nonparticipant groups, adjusting for the following variables: patient age (0 – 1 yr, 2 – 5 yr, 6+ years), sex (male, female), race/ethnicity (White, Black, Hispanic/Latinx, Other), insurance type (commercial, governmental), prescription order date, provider type (physician, nurse practitioner, trainee), providers’ baseline AOM prescribing (continuous variable of percent guideline-concordant prescribing during the baseline period), and clinic type (pediatric urgent care, adult/pediatric urgent care, emergency medicine, primary care, and retail). Statistical analyses were performed using R Statistical Software (v4.4.1). Due to few diagnoses of GAS, CAP, and UTI, only guideline-concordant prescribing for AOM was analyzed (Figure 1).

## Results

In total, 52 prescribers exclusively participated in the basic intervention, 10 participated in the enhanced intervention, and 301 did not participate in either (Figure 1). Of the basic intervention participants, 38 of 52 (73.1%) completed at least 9 out of 10 questions. Of the enhanced intervention participants, 7 of 10 (70.0%) completed all 5 questions. In total, 15 306 AOM prescriptions were ordered during the baseline period, 6 044 during period 1, 9 167 during period 2, and 8 447 during the sustainability period. Among nonparticipants there was no statistically significant difference in guideline-concordant durations between study periods. The maximum median change from baseline per provider for AOM age-appropriate duration occurred in period 2 for all groups. Guideline-concordant duration increased among nonparticipants by 0.5%, among basic intervention participants by 10.0%, and among enhanced intervention participants by 13.0% (*P* = .04). During the sustainability period, guideline-concordant prescribing remained above baseline but fell for both groups; no statistical significance was observed between the groups (*P* = .3).

**Figure 2.**
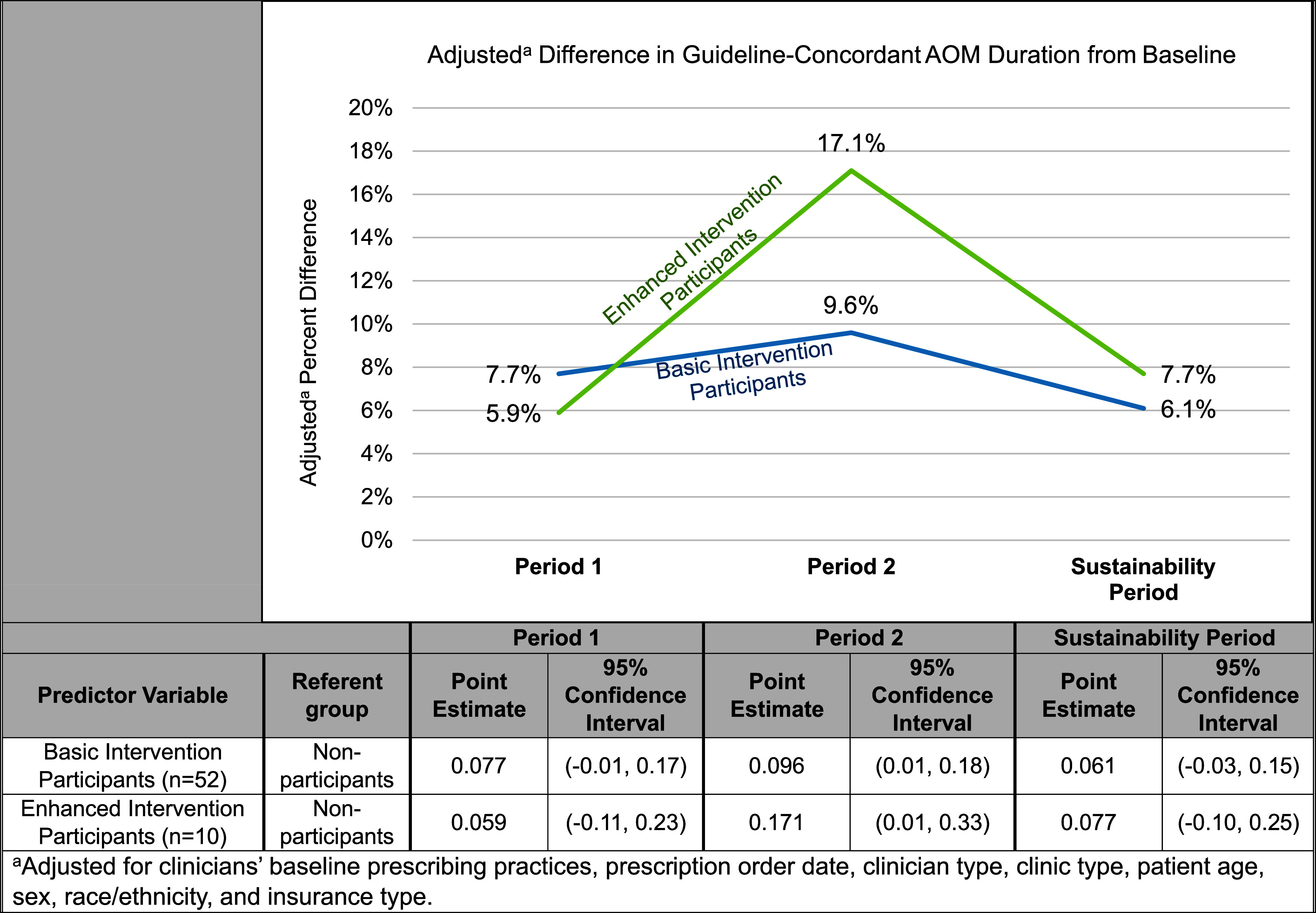
Percent adjusted difference in guideline-concordant prescriptions for AOM duration between participants and nonparticipants.

Similar results were seen in our multivariate logistic regression analysis after adjusting for the clinicians’ baseline AOM prescribing practices, clinician type, clinic type, and the patient’s age, sex, race/ethnicity, and insurance type. Participants in either intervention group had a statistically significant improvement in guideline-concordant prescribing compared to nonparticipants during period 2 (Figure 2). Participants in either intervention group did not show a statistically significant change from baseline during period 1 or the sustainability period.

## Discussion

This prospective cohort study found that clinician participation in a microlearning web-based quiz improved prescribing of guideline-concordant antibiotic treatment duration for AOM. After adjusting for several covariates including clinician’s baseline prescribing practices, participants had improved guideline-concordant prescribing when compared to nonparticipants, demonstrating that QuizTime may be a tool that can successfully disseminate educational content about guideline-concordant antimicrobial prescribing with minimal stewardship effort. Consistent with prior studies and reflected in our logistic regression model, improvements in prescribing were not sustained after quizzes stopped,^8^ emphasizing the importance of ongoing educational initiatives. We hypothesize that low enrollment in the basic intervention may be due to a limited marketing campaign during the summer when AOM rates are lower. This then led to a small sample size for the enhanced intervention as we chose to invite only half of the basic intervention participants. This study has several limitations. Participation was voluntary and therefore participants likely differed from nonparticipants. The small sample size in the enhanced intervention limits power and generalizability. While questions covered AOM, CAP, UTI, and pharyngitis testing, we could only assess AOM prescriptions due to limited number of prescriptions for other diagnoses. Additionally, other outpatient antimicrobial stewardship activities, such as dissemination of a quick-reference guide for common pediatric infections, were ongoing during the study. However, this guide was provided to both participants and nonparticipants, and no change was observed in the nonparticipant group, suggesting that QuizTime had additional impact.

In conclusion, our study demonstrates that exposure to microlearning quizzes was associated with a 10% increase in guideline-concordant antibiotic duration for AOM but was not sustained when quizzes stopped. Additionally, exposure to multiple quizzes showed a trend towards greater effect, although larger studies are needed to confirm this finding.

## Supporting information

10.1017/ash.2025.10160.sm001 Lehrer et al. supplementary material Lehrer et al. supplementary material

## Supplementary material

The supplementary material for this article can be found at https://doi.org/10.1017/ash.2025.10160.
