# Supplementary material for: It’s QuizTime! The impact of web-based microlearning quizzes on guideline-concordant antibiotic duration for pediatric acute otitis media
Source: Antimicrob Steward Healthc Epidemiol. 2025 Nov 3;5(1):e295. doi: 10.1017/ash.2025.10160 (PMC12616573; doi:10.1017/ash.2025.10160)
Supplement: Lehrer et al. supplementary material [file S2732494X25101605sup001.docx]

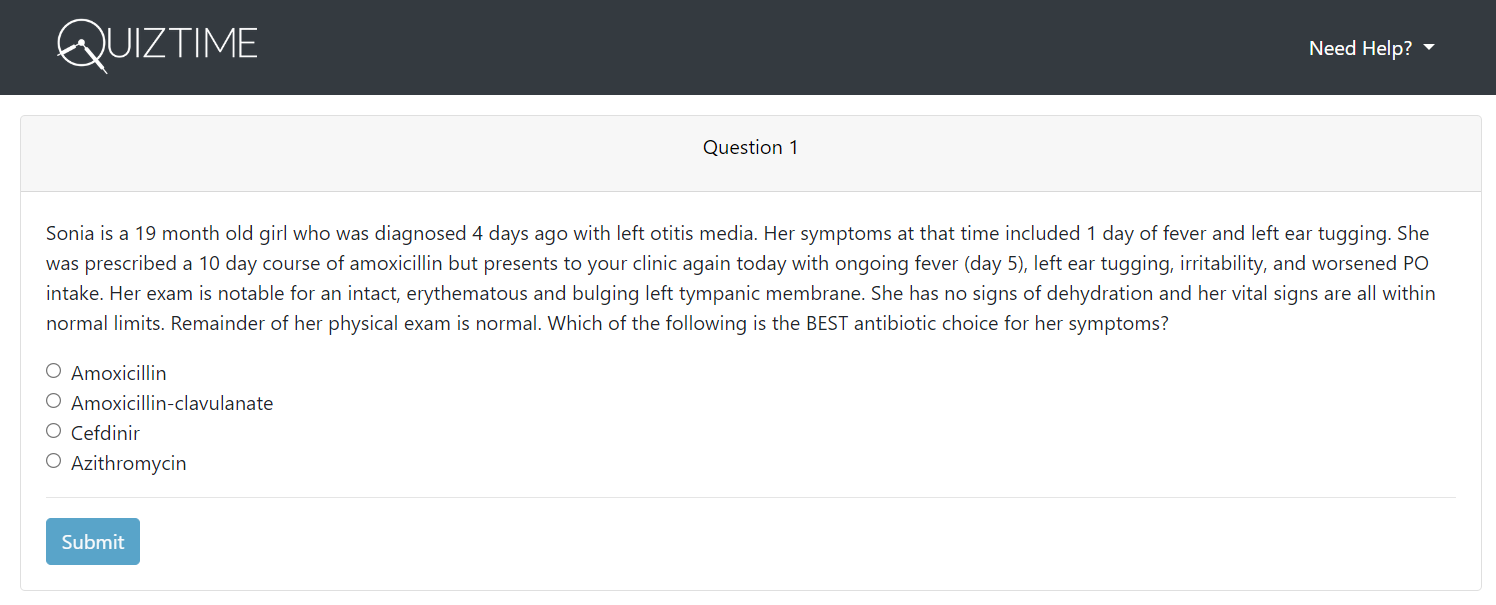


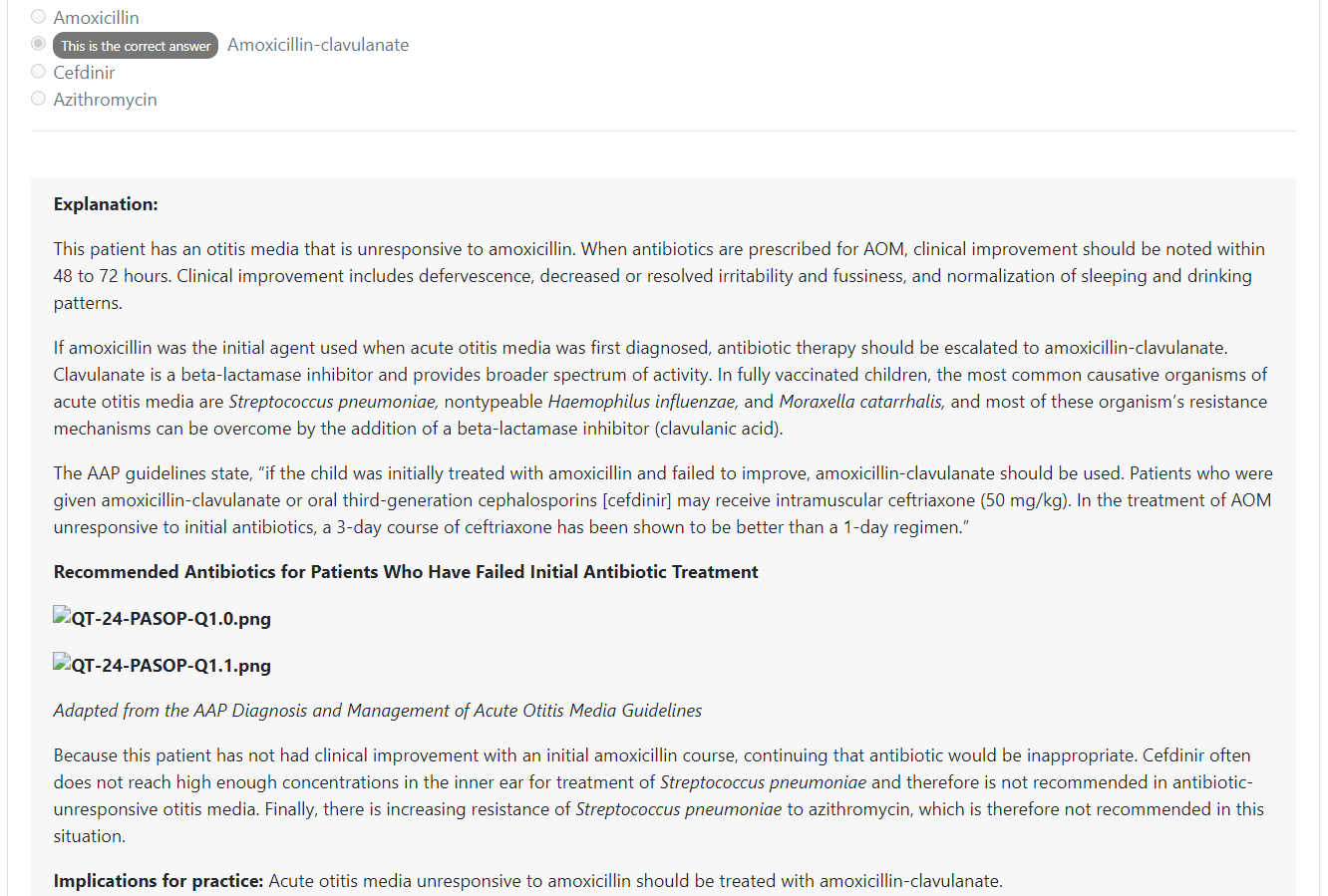


Supplemental Figure 1: An example of an answer with learning objectives from one of the QuizTime case-based questions in the basic intervention.

**Baseline Period**

Baseline data collected

July 2021 – June 2022

**Q1**

**Q2**

**Q3**

**Q4**

**2021**

**Basic Intervention**

(2-weeks; 10-questions)

Enrollment July 1 – Oct 15 2022


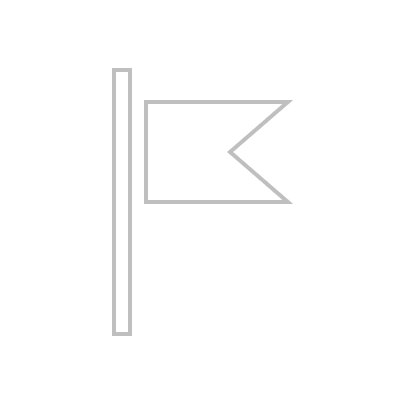


01

**Q1**

**Q2**

**Q3**

**Q4**

**2022**

**Enhanced Intervention**

(1-week; 5-questions)

Enrollment November 1 2022 – Mar 21, 2023


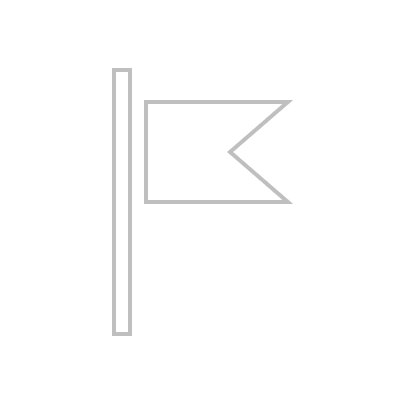


02

**Q1**

**Q2**

**Q3**

**Q4**

**2023**

**Period 1**

Basic Intervention data

July 2022 – Oct 2022

**Period 2**

Enhanced Intervention data

Nov 2022 – Mar 2023

**Sustainability Period**

Post-Intervention data

Apr 2023 – Sept 2023

**Interventions**

- **Basic Intervention**: Could be taken by any clinician in the outpatient setting
- **Enhanced Intervention**: Clinicians who participated in the Basic Intervention were randomized to possibly receive the Enhanced Intervention

**Comparator groups**

- Non-participants (control)
- Participants of the Basic Intervention (exclusively)
- Participants of the Enhanced Intervention

Supplemental Figure 2: Outline of associated time periods, interventions, and comparator group.

Supplemental Methods: All QuizTime Questions with Answers

**QUESTION #1 in the basic intervention; QUESTION #1 in the enhanced intervention:**

**TOPIC:** Duration of therapy for acute otitis media

JG is a 6-year-old boy with no significant past medical history who presents to your urgent care clinic with 1 day of right-sided ear pain and fever to 101 degrees Fahrenheit. On physical exam, you note mild bulging and erythema of the right tympanic membrane. The remainder of his exam was normal. You choose to prescribe amoxicillin 90mg/kg/day to treat his ear infection. Which of the following is the BEST duration of antibiotics?

1. 3 days
2. **5 days**
3. 10 days
4. 14 days

**Explanation:**

Antibiotics have side effects. The longer a person is exposed to an antibiotic, the more likely they are to develop side effects. Antibiotic exposure also increases risk of bacterial resistance. Using the shortest course of an antibiotic that will effectively treat an infection is considered best practice. For acute otitis media (AOM), studies have shown that children younger than 2 years have better outcomes with longer antibiotic durations. Older children, however, can safely be treated with shorter antibiotic courses. The 2013 AAP guidelines recommend the following durations for children with mild or moderate acute otitis media:

- Children < 2 years, 10-days of antibiotics has been shown to have more benefit than shorter courses.
- Children 2 to 5 years: 7-day course appears to be equally as effective as longer courses
- Children 6 years and older: 5- to 7-day course is adequate treatment

Children of any age with severe signs warrant longer courses (10-days) of antibiotics. Severe signs include fever > 39 degrees Celsius (102.2 degrees Fahrenheit) or moderate to severe otalgia or otalgia > 48 hours.

**Image:** AAP’s Education in Quality Improvement for Pediatric Practice (EQIPP) AOM flowchart

**Implications for practice:** For children with mild or moderate acute otitis media, the AAP recommends antibiotic durations based on age:

- Children < 2 years: 10-days
- Children 2 to 5 years: 7-days
- Children > 6 years: 5-days

**References:**

- Liberthal et al. AAP Clinical Practice Guidelines: the diagnosis and management of acute otitis media. *Pediatrics*, 2013; 131(3): e964-e999.
- AAP EQIPP module
- Pichichero et al. A prospective observational study of 5-, 7- and 10-day antibiotic treatment for acute otitis media. *Otolaryngol Head Neck Surg*, 2001; 124(4):381-7.

**QUESTION #2 in the basic intervention:**

**TOPIC:** Testing for Group A Streptococcal pharyngitis

RB is a previously healthy 7-year-old girl who presents to clinic with 1-day history of sore throat and fever to 102 degrees Farenheit. She denies cough, congestion or rhinorrhea. On physical exam, you note enlarged and erythematous tonsils with white exudate and palatal petechiae. Which of the following tests is most likely to lead to her diagnosis?

1. Rapid influenza test
2. Rapid RSV test
3. **Rapid Strep test**
4. Viral respiratory PCR (RPP)

**Explanation:**

Viruses are the most common cause of pharyngitis in children. Group A strep accounts for only 20- 35% of pharyngitis episodes in children. However, an estimated 60% of children with pharyngitis receive an antibiotic indicating that many children with pharyngitis are unnecessarily prescribed antibiotics.

Rapid strep tests have notoriously high false-positive rates, due to detection of Group A Strep carriers, variability in interpretation of results (that pink line can be hard to read!), or because of poor test performance of the rapid antigen tests. Testing for strep in children is indicated for those who have a high likelihood of having streptococcal infection, but not for children with viral syndromes. Criteria for children in whom a strep test is appropriate are outlined in the table below. Additionally, whether a strep test was obtained before prescribing antibiotics for streptococcal pharyngitis is a metric tracked in the Healthcare Effectiveness Data and Information Set (HEDIS), a healthcare performance improvement tool often tied to reimbursement.

| **GAS Pharyngitis Testing Criteria** |
| --- |
| Patient age > 3 years (unless household contact with confirmed GAS pharyngitis) |
| Presence of sudden onset sore throat, generally with fever |
| Absence of cough, hoarseness, coryza, conjunctivitis, viral exanthem, mouth ulcers, diarrhea (i.e. viral symptoms) |
| Presence of 1 or more of the following:   - Tonsillopharyngeal erythema - Patchy tonsillopharyngeal exudates - Palatal petechiae - Sudden onset of sore throat and fever - Tender anterior cervical adenopathy - Scarlatiniform rash - Swollen red uvula |

**Implications for practice:** Do not test for GAS pharyngitis in children < 3 years or those with viral symptoms.

**References:**

- AAP EQIPP module
- Dooling KL et al. Overprescribing and inappropriate antibiotic selection for children with pharyngitis in the United States, 1997-2010. *JAMA Pediatrics*, 2014; 168(11):1073-1074.
- Kronman MP et al. Bacterial prevalence and antimicrobial prescribing trends for acute respiratory tract infections. *Pediatrics,* 2014; 134(4):e956.
- Shulman ST et al. Clinical practice guideline for the diagnosis and management of group A streptococcal pharyngitis: 2012 update by the Infectious Diseases Society of America. *Clinical Infectious Diseases,* 2012; 55(10):e86.

**QUESTION #3 in the basic intervention:**

**TOPIC:** First-line antibiotic for community acquired pneumonia

SS is a 3-year-old girl with past medical history of acute otitis media s/p tympanostomy tube placement at age 2 who presents to clinic with 4 days of rhinorrhea, cough, and congestion and 1 day of fever and difficulty breathing. On physical exam, you note an uncomfortable but well-appearing child with mild subcostal retractions and focal crackles in the right lower lung field. Oxygen saturation is 95% in room air. You suspect bacterial pneumonia. Because she is well-appearing, you elect to treat with a 5-day course of oral antibiotics as an outpatient. What is the BEST antibiotic choice?

1. **Amoxicillin**
2. Azithromycin
3. Cefdinir
4. Levofloxacin

**Explanation:**

The most common causes of pneumonia in a 3-year-old child are viruses (even in acute lobar pneumonia). If bacterial pneumonia is suspected, the most common etiology is *Streptococcus pneumoniae*. Atypical pathogens such as *Mycoplasma* are uncommon in children younger than 5 years and there is conflicting data regarding the benefit of treatment of *Mycoplasma* pneumonia.

According to VUMC’s antibiogram, in 2021, 93% of *Streptococcus pneumoniae* isolates were susceptible to amoxicillin but only 50% were susceptible to azithromycin (see table below). Thus, amoxicillin and ampicillin remain the drugs of choice to treat uncomplicated community-acquired pneumonia.

Cefdinir and levofloxacin are excessively broad-spectrum antibiotic choices (cover too many Gram-negative bacteria) and thus inappropriate options. Additionally, cefdinir does not have good lung penetration with doses approved by the FDA and therefore is not a good choice to treat pneumonia. Children with pneumonia who improve on cefdinir may have had viral pneumonia.

**Image:** VUMC Antibiogram with pediatric *S. pneumoniae* isolates and associated antibiotics susceptibilities.

**Implications for practice:** Amoxicillin is the preferred antibiotic choice for uncomplicated community-acquired pneumonia in children.

**References:**

- Jain S et al. Community-acquired pneumonia requiring hospitalization among U.S. children. *The New England Journal of Medicine*, 2015; 372:835-45.
- Bradley et al. The management of community-acquired pneumonia in infants and children older than 3 months of age: clinical practice guidelines by the Pediatric Infectious Diseases Society and the Infectious Diseases Society of America. *Clinical Infectious Diseases*, 2011; 57(3): e25-76.

**QUESTION #4 in the basic intervention; QUESTION 2 in the enhanced intervention:**

**TOPIC:** First-line antibiotic for pediatric urinary tract infections

KB is a 8-year old girl with a past medical history of constipation who presents to urgent care with 1-day of dysuria and urinary frequency. Tmax is 100.2 and physical exam is notable for suprapubic tenderness. Her urine dipstick is notable for nitrites, and leukocyte esterase. You decide to empirically prescribe an antibiotic for a urinary tract infection while awaiting culture results. What is the BEST empiric antibiotic choice?

1. Amoxicillin
2. **Cephalexin**
3. Cefdinir
4. Trimethoprim-sulfamethoxazole

**Explanation:**

*Escherichia coli* accounts for most uncomplicated pediatric urinary tract infections (UTI; about 65-70% each year). So, it is the best bacteria to target with empiric antibiotic treatment for UTI. According to the 2021 antibiogram, 92% of the *E. coli* isolates were susceptible to cephalexin. Cephalexin also has good penetration into the kidneys, and is therefore a good choice for treating pyelonephritis as well.

A commonly prescribed antibiotic is cefdinir and about 95% of isolates were susceptible to it on the 2021 antibiogram, but cefdinir is more broad-spectrum than cephalexin, so it is not the preferred choice. Empiric antibiotics for children with a complex urologic history or a history of recurrent UTIs should be based on prior culture data.

**Image**: VUMC Pediatric Antibiogram with most common urinary pathogens and associated antibiotic susceptibilities.

**Implications for practice:** Cephalexin or nitrofurantoin are the preferred empiric antibiotic choices for uncomplicated urinary tract infections in children.

**References:**

- Vanderbilt Children’s Hospital Urinary Tract Infection Clinical Practice Guideline – available at <https://www.vumc.org/childrens-quality-safety/clinical-practice-guidelines-urinary-tract-infection>

**QUESTION #5 in the basic intervention:**

**TOPIC:** Antibiotic-related adverse events

MA is a 1-year-old boy who was started on cefdinir for an upper respiratory tract infection by an urgent care physician while the family was on vacation in Florida 4 days ago. He presents to your office with a rash that started 2 days ago. On exam, the rash is non-specific, erythematous, maculopapular, and not pruritic. The boy is otherwise well appearing and at his baseline energy level. He is afebrile and has mild rhinorrhea, but the remainder of his physical exam is normal. Which of the following is the next BEST step in his management?

1. Send him to the emergency department for treatment of anaphylaxis
2. **Stop his cefdinir and tell him he likely had a viral illness**
3. Stop his cefdinir and record it as an allergy in his chart. Prescribe azithromycin instead.
4. Stop his cefdinir and record it as an allergy in his chart, but do not prescribe any alternative antibiotics.

**Explanation:**

Adverse drug events from antibiotics lead to about 70,000 ED visits per year in the United States. Most of these visits occur in children younger than 2 years old, with rash and gastrointestinal upset being the two most common reasons for presentation.

Viral upper respiratory tract infections account for most illnesses in children because they are being exposed to many viruses for the first time. A non-specific maculopapular rash is a common finding in pediatric viral URI, and sometimes can occur days into the illness. A non-specific maculopapular rash is not an indication of anaphylaxis. In a child with viral symptoms, a non-specific maculopapular rash is more likely to be from a virus than from an adverse drug event.

**Implications for practice:** Antibiotics are not benign and side effects are common.

**References:**

- Lovegrove et al. US emergency department visits for adverse drug events from antibiotics in children, 2011-2015. *Journal of the Pediatric Infectious Diseases Society*, 2018; 8(5):384-391.
- Dialogue Around Respiratory Illness Treatment (DART): <https://www.uwimtr.org/dart/>
- Fleming-Dutra KE et al. How to prescribe fewere unnecessary antibiotics: talking points that work with patients and their families. *Am Fam Physician*, 2016; 94(3):200-2.
- Mangione-Smith et al. Communication practices and antibiotic use for acute respiratory tract infections in children. *Ann Fam Med*, 2015; 13(3):221-7.

**QUESTION #6 in the basic intervention; QUESTION 3 in the enhanced intervention:**

**TOPIC:** Guideline-recommended treatment duration for pneumonia

A 4-year-old girl presents to clinic with 5 days of cough and congestion and 2 days of fever (Tmax 101.2) and increased work of breathing. On physical exam, her heart rate is 110, respiratory rate is 35, and oxygen saturation is 92% in room air. You note crackles in her right lower lobe. Because she is overall well-appearing, you elect to treat her as an outpatient. She has no known antibiotic allergies. What antibiotic choice and duration is the BEST option to prescribe to treat her illness?

1. Amoxicillin 90mg/kg/dose divided q12h for 10 days
2. **Amoxicillin 90mg/kg/dose divided q12h for 5 days**
3. Cefdinir 15mg/kg/dose q12h q24h for 5 days
4. Cefdinir 15mg/kg/dose q12h for 10 days

**Explanation:**

This child’s presentation is consistent with a viral URI complicated by possible bacterial pneumonia. The most common bacterial cause of community-acquired pneumonia is *S. pneumoniae*, and the best antibiotic to treat uncomplicated community-acquired pneumonia is amoxicillin, to which 93% of pediatric *S. pneumoniae* isolates remain susceptible based on the VUMC antibiogram.

The recommended antibiotic duration for uncomplicated outpatient community-acquired pneumonia has changed based on 2 research studies published in 2021 and 2022. These trials demonstrate that it is safe and effective to use short courses of antibiotics. In fact, a 5-day treatment course was shown to be SUPERIOR to a standard 10-day course,

The AAP Red Book contains a systems-based treatment table that summarizes evidence-based antibiotic choice and duration for common pediatric infectious syndromes, including community-acquired pneumonia.

**Implications for practice:** 5-days of antibiotics are adequate for treating uncomplicated community-acquired pneumonia.

**References:**

- AAP Red Book Systems-based Treatment Table
- Bielicki JA et al. Effect of amoxicillin dose and treatment duration on the need for antibiotic re-treatment for children with community-acquired pneumonia: the CAP-IT randomized clinical trial. *JAMA*, 2021; 326(17): 1713-1724.
- Williams DJ et al. Short- vs standard-course outpatient antibiotic therapy for community-acquired pneumonia in children: The SCOUT-CAP randomized clinical trial. *JAMA Pediatrics*, 2022; 176(3):253-261.

**QUESTION #7 in the basic intervention; QUESTION #4 in the enhanced intervention:**

**TOPIC:** Strep pharyngitis testing

A 2-year-old boy presents to your office in late May with a chief complaint of sore throat. On history, you elicit that he has had 2 days of cough, congestion, rhinorrhea, fever to 101 degrees Fahrenheit, as well as sore throat. He has no sick contacts at home but does attend daycare. The parents are especially concerned about Strep throat and are requesting an antibiotic. Physical exam is notable for an erythematous posterior pharynx and mildly enlarged tonsils without exudate. There is fluid behind his bilateral tympanic membranes, but no erythema or bulging. What is the NEXT BEST step in management of this child?

1. Order a rapid strep test
2. Order a rapid influenza test
3. Provide an antibiotic prescription
4. **Provide reassurance**

**Explanation:**

This child most likely has a viral upper respiratory tract infection. The next best step in his management is to provide reassurance, including (1) a tangible diagnosis (viral URI), (2) positive treatment recommendations like symptomatic care (Tylenol, fluids, rest, etc) and (3) a contingency plan for the family to follow if his symptoms are not improved in the next 3 days.

It can be uncomfortable to have a conversation about antibiotics with a family who expects antibiotic treatment and sometimes providers feel it is easier to simply prescribe an unwarranted antibiotic. There are, however, methods of communication that can help in this instance. One research-proven method is the Dialogue Around Respiratory Illness Treatment (DART) (<https://www.uwimtr.org/dart/>). This communication tutorial consists of 7 modules, all 5 minutes or shorter, to equip providers with skills to address families when faced with a situation where unwarranted antibiotics may otherwise be prescribed.

While this patient has erythema of his posterior pharynx on exam, his young age (< 3) and presence of URI symptoms make Group A Strep pharyngitis an unlikely diagnosis. We treat strep to prevent complications such as acute rheumatic fever, which is rare in the United States. Furthermore, the risk of acute rheumatic fever in children younger than 3 years is very rare.

The seasonality (summer) makes influenza unlikely. Ordering either a rapid strep or influenza test could either result in a false-positive result or a negative result, and thus those answer options are incorrect.

**Implications for practice:** Viruses are the most common cause of pharyngitis in children and do not require antibiotic treatment.

**References:**

- Dialogue Around Respiratory Illness Treatment (DART): <https://www.uwimtr.org/dart/>
- Shulman ST et al. Clinical practice guideline for the diagnosis and management of group A streptococcal pharyngitis: 2012 update by the Infectious Diseases Society of America. *Clinical Infectious Diseases,* 2012; 55(10):e86.
- Woods WA, Carter CT and Schlager TA. Detection of group A streptococci in children under 3 years of age with pharyngitis. *Pediatr Emerg Care*, 1999; 15(5):338-340.

**QUESTION #8 in the basic intervention:**

**TOPIC:** Antibiotic-related adverse events

**Learning Objective Map #**: 2,3

AK is a 5-year-old girl who is being treated with an antibiotic for a urinary tract infection. Four days into the course of her treatment, she presents to the clinic with fever, flu-like symptoms and a painful red rash and blisters on her lip. On physical exam, you note an uncomfortable appearing child with erythematous and blistering macules on her bilateral lower extremities and blistering of her lips and mouth. You appropriately refer her to the emergency department for further care. Which antibiotic is the MOST LIKELY cause of her symptoms?

1. Cefdinir
2. Cephalexin
3. Doxycycline
4. **Trimethoprim-sulfamethoxazole**

**Explanation:**

This patient has Stevens-Johnson Syndrome (SJS). In children, medications are the most common precipitant of SJS. Of the antibiotic options listed, the most likely cause is trimethoprim-sulfamethoxazole.

SJS manifests as fever and flu-like symptoms with involvement of skin and mucosa arising 1 to 3 days later. Ophthalmic complications also present in up to 30% of patients. Management largely involves withdrawal of offending agents and aggressive supportive care (wound care, fluid and electrolyte management, nutritional support, temperature management, ocular care, pain control and pulmonary toilet). Patients should be referred to the emergency department for this supportive care.

In a study by Chan et al. evaluating records of patients hospitalized with erythema multiforme, Stevens Johnson syndrome or toxic epidermal necrolysis, the most frequent drugs found to cause these disorders included phenobarbital (20 per 100,000 exposed individuals), nitrofurantoin (7 per 100,000), trimethoprim-sulfamethoxazole and ampicillin (both 3 per 100,000) and amoxicillin (2 per 100,000). These findings highlight that medication use is not always benign and provide an example of why judicious antibiotic use is important. This case also highlights why cephalexin is the preferred treatment for uncomplicated pediatric urinary tract infections.

**Implications for practice:** Antibiotics are not benign and side effects are common.

**References:**

- Alerhand S, Cassella C and Koyfman A. Stevens-Johnson syndrome and toxic epidermal necrolysis in the pediatric population: A review. *Pediatric Emergency Care*, 2016; 32(7):472-476.
- Chan HL et al. The incidence of erythema multiforme, Stevens-Johnson syndrome, and toxic epidermal necrolysis. A population-based study with particular reference to reactions caused by drugs among outpatients. *Arch Dermatol*, 1990; 126(1):43-47.

**QUESTION #9 in the basic intervention; QUESTION #5 in the enhanced intervention:**

**TOPIC:** Duration of therapy for acute otitis media

KG is a 1-year-old boy who presents to clinic with 2 days of fever to 101 F and pulling on his right ear. He also has congestion, cough, and rhinorrhea. On physical exam, you note an erythematous and bulging right tympanic membrane. The remainder of his physical exam is normal. You decide to prescribe amoxicillin 90mg/kg/dose. What is the BEST duration of antibiotics?

1. 3 days
2. 5 days
3. **10 days**
4. 14 days

**Explanation:**

While older children with uncomplicated acute otitis media can be treated with shorter durations of antibiotics (5-7 days), there is evidence that children < 2 have better outcomes with a 10-day course. In a trial where children were randomized to receive either 10-days of amoxicillin-clavulanate or 5-days of amoxicillin-clavulanate followed by 5-days of placebo, those in the short-course group were more likely to have clinical failure than those in the long-course group. The AAP guidelines recommend a 10-day treatment course for children under 2 years.

The 2013 AAP guidelines recommend the following durations for children with mild or moderate acute otitis media:

- Children < 2 years, 10-days of antibiotics has been shown to have more benefit than shorter courses.
- Children 2 to 5 years with mild or moderate AOM: 7-day course appears to be equally as effective as longer courses
- Children 6 years and older with mild to moderate symptoms: 5- to 7-day course is adequate treatment

No matter the age, children with severe signs warrant longer courses (10-days) of antibiotics. Severe signs include fever > 39 degrees Celsius (102.2 degrees Fahrenheit) or moderate to severe otalgia or otalgia > 48 hours.

**Image:** AAP’s Education in Quality Improvement for Pediatric Practice (EQIPP) AOM flowchart.

**Implications for practice:** Children < 2 years with acute otitis media should be treated with 10-days of antibiotics.

**References:**

- Hoberman A et al. Shortened antimicrobial treatment for acute otitis media in young children. *New England Journal of Medicine*, 2016; 375:2446-2456.
- Liberthal et al. AAP Clinical Practice Guidelines: the diagnosis and management of acute otitis media. *Pediatrics*, 2013; 131(3): e964-e999.
- AAP EQIPP module

**QUESTION #10 in the basic intervention:**

**TOPIC:** Treatment of pediatric urinary tract infections in a patient with cephalexin allergy

KB is an 8-year-old girl with a past medical history of constipation and urinary tract infections who presents to urgent care with 1-day of dysuria and increased urinary frequency. She is afebrile and does not endorse flank pain. Physical exam is notable for suprapubic tenderness. Her urine dipstick is notable for nitrites and leukocyte esterase. You decide to empirically prescribe an antibiotic while awaiting culture results. She reports a history of hive-like rash when she took cephalexin for her UTI last year. She can swallow pills. What is the BEST empiric antibiotic choice?

1. Amoxicillin
2. Ciprofloxacin
3. Levofloxacin
4. **Nitrofurantoin**

**Explanation:**

The most common bacteria to cause uncomplicated urinary tract infections is *E. coli*. According to local antibiogram data and our local urinary tract infection CPG, the best empiric antibiotic choice for uncomplicated UTI (cystitis) is cephalexin. This patient, however, has a history of hives with cephalexin which could represent a true allergy. The next best choice is therefore nitrofurantoin.

Nitrofurantoin does not penetrate the renal parenchyma; therefore it is a suboptimal choice for treatment of pyelonephritis. However, this patient does not have clinical signs of pyelonephritis. Nitrofurantoin suspension can be expensive, so be sure to check with the patient first (ask about insurance coverage/cost and if they can swallow pills) or only prescribe capsules. The monohydrate/macrocrystals capsules (twice-daily formulation [eg, Macrobid]) should not be opened for mixing the contents with food or juice; the macrocrystals capsules (4-times-daily formulation [eg, Macrodantin]) may be opened and the contents mixed with food or juice for immediate use. If four-times-daily prescribing would be difficult for the family, it is reasonable to prescribe a fluroquinolone (ciprofloxacin) for empiric therapy.

All patients with suspected UTI should have a urine culture sent and antibiotics should be altered based on final culture identification and susceptibility.

**Image**: VUMC Pediatric Antibiogram with most common urinary pathogens and associated antibiotic susceptibilities.

**Implications for practice:** Cephalexin or nitrofurantoin are the preferred empiric antibiotic choices for uncomplicated urinary tract infections in children.

**References:**

- Lexicomp: Nitrofurantoin. Accessed via web on 4/7/2022 (<https://online.lexi.com/lco/action/doc/retrieve/docid/vanderbiltchild_f/5606148?cesid=0RgNwZaKUNF&searchUrl=%2Flco%2Faction%2Fsearch%3Fq%3Dnitrofurantoin%26t%3Dname%26va%3Dnitrofurantoin>)
- Vanderbilt Children’s Hospital Urinary Tract Infection Clinical Practice Guideline – available at <https://www.vumc.org/childrens-quality-safety/clinical-practice-guidelines-urinary-tract-infection>
